# Sperm performance in the race for fertilization, the influence of female reproductive fluid

**DOI:** 10.1098/rsos.240156

**Published:** 2024-07-31

**Authors:** Livia Pinzoni, Maria Berica Rasotto, Clelia Gasparini

**Affiliations:** Department of Biology, University of Padova, Padova 35131, Italy

**Keywords:** fertilization, female reproductive fluid, ovarian fluid, sperm performance, sperm competition

## Abstract

In studies of sperm competition, particularly in external fertilizers, the importance of the fertilization environment on the paternity share among rival males often goes overlooked. The female reproductive fluid (FRF), produced and released by females, creates the microenvironment that sperm encounter on their quest for fertilization and can generate paternity biases by affecting key traits in sperm competition. Yet, whether there is a direct link between FRF effects on sperm traits and its effect on competitive fertilization dynamics remains to be explored. Here, using the zebrafish *Danio rerio*, we compare within-female paternity share among two competing males and predictors of fertilization success (i.e. sperm traits) in the presence/absence of FRF. Our results unequivocally reveal a direct link between the direction and magnitude of the effect of FRF on sperm traits and the change in the competitive fertilization success of each male. This study demonstrates that the FRF directly mediates post-mating female control through its differential effect on sperm performance and that the FRF's effect on sperm quality alone is sufficient to predict the magnitude of the fitness effects. These findings highlight the need to consider the role of FRF in fertilization, avoiding biases resulting from an exclusive focus on male intrinsic sperm quality.

## 1. Introduction

Fertilization often entails competition among sperm from multiple males to fertilize the same set of eggs. The outcome of competitive fertilization has traditionally been ascribed to the relative differences in the intrinsic characteristics of the ejaculate among rival males, encompassing sperm number and quality [1–5]. Despite the increasing knowledge of the processes mediating sperm competition (i.e. the competition among ejaculates from different males to fertilize the same set of eggs; [6]), it is unlikely that fertilization outcomes can be inferred only by the relative differences in sperm number or quality among the competitors. This is not surprising, as fertilization occurs in an environment strongly influenced by the female, and female contribution cannot be forgotten. In internal fertilizers, the arena in which sperm compete is indeed vastly affected by the interaction of the ejaculates with the environment created by the female reproductive tract and its derivatives [7]. This interaction often results in a paternity bias, a process known as cryptic female choice (CFC, [8,9]). Although CFC has traditionally been studied in internally fertilizing species [10], it is deemed to play an important selective role in external fertilizers due to the limited pre-mating choice opportunities for females of these species compared to their internally fertilizing counterparts.

A shared mechanism that females of both internally and externally fertilizing species may use to influence the fertilization process is the female reproductive fluid. The term female reproductive fluid (hereafter, FRF) refers to any medium, produced by females, through which sperm must pass on their way to fertilize eggs [11]. This fluid is ubiquitously present during fertilization; it is kept inside the female reproductive tract in the case of internal fertilizers, or released in the external environment, whenever eggs are fertilized outside the female body. Besides playing a crucial role in oocyte maturation, egg viability, fertilization, and early embryo development [12–15] FRF affects key sperm behaviour parameters essential for successful fertilization, including viability, chemotaxis, longevity and swimming velocity and trajectory (e.g. [16–21]). Recent evidence has identified FRF as a mediator of cryptic female choice, allowing females to influence the outcome of sperm competition by differentially affecting sperm of the competing males. FRF has been reported to have differential effects based on male by female interactive effects on sperm performance in a variety of species, spanning across diverse taxa and fertilization modes (e.g. [5,19,22–26]) and, ultimately, to favour fertilization towards phenotypically or genetically preferred males [27–30]. Although evidence supporting the role of FRF on fertilization outcome is increasing, to date no experiment has been able to examine whether, and to what extent, the direction and magnitude of FRF effects on sperm performance might directly translate into a paternity bias. Here, we use a split clutch approach (of eggs and ejaculates) (i) to perform *in vitro* fertilization both in the presence and absence of FRF within the same female, (ii) we standardize sperm number among males, to focus on the effect on sperm quality, and (iii) we combine in the same experiment sperm performance measurements and paternity outcome both in the presence and absence of FRF. While previous research has demonstrated the potential for FRF to influence paternity outcomes also in external fertilizers [29,31], our study represents the first application of such a multi-layered approach to this context. Through this novel approach, we were able to unequivocally reveal the direct link between FRF effects on sperm performance and its impact on paternity outcome.

Using an external fertilizer to understand the strength of the selection imposed by FRF at the post-mating stage is ideal for several reasons. The *in vitro* fertilization technique allows the recreation of a fertilization environment for sperm and eggs that is similar to what is experienced under natural conditions, and allows us to exclude any possible behavioural interaction between males and females and thus focus on post-mating dynamics. Using an external fertilizer also provides the opportunity to use the split-clutch approach, to estimate paternity bias within the same egg clutch and at the same time minimize other confounding variables. We used the zebrafish *Danio rerio* as model species, building on previous knowledge of the FRF effects on sperm and egg traits [15,24,32,33]. The zebrafish is being increasingly appreciated as a suitable model for reproduction and sexual selection dynamics. In the wild, females spawn in shallow water in the presence of one or more males [34,35], leading to broods with multiple paternity [36]. Importantly, Poli *et al*. [24] provided evidence of the differential effects of FRF on sperm traits, indicating that it could have the potential to facilitate sperm selection.

We investigated whether male–female interactive effects on sperm performance generated by the FRF can directly and proportionally promote paternity biases. Specifically, we were interested in exploring whether the direction and magnitude of the fertilization shift in the presence of FRF is determined by the parallel shift in sperm traits mediated by the FRF. To do so, we assessed and compared the paternity share and predictors of fertilization success (sperm motility, swimming velocity, trajectory and longevity) between two competing males under the same conditions (in the presence and absence of FRF). This approach enables us to disentangle male- and female-mediated effects in post mating dynamics, to explore whether intrinsic differences between males predict competitive fertilization success (still unknown in this species), and to simultaneously test whether FRF allows females to potentially override the relative intrinsic quality of sperm to ultimately bias fertilization in their own interest.

## 2. Material and methods

### 2.1. Fish maintenance

Zebrafish used in this study were Tuebingen wild type, reared under standard laboratory conditions at the Zebrafish Facility of the Department of Biology (University of Padova, Italy). All fish were kept in mixed-sex groups of 15 fish in 3 L tanks in a recirculating rack system (Tecniplast) at a water temperature of 28 ± 1°C with a 12L : 12D photoperiod and fed ad libitum three times per day with a mix of dry food and *Artemia salina* nauplii. Adult males and females were allowed to spawn every week in groups of 6–8 fish with a 50 : 50 sex ratio to preserve optimal reproductive health. Before the experimental procedure males and females were kept separated for a week using a transparent barrier that allowed both olfactory and visual stimuli. Both males and females used for the experiments were 9–11 months old.

### 2.2. Overview of the experimental design

We tested whether the presence of FRF affects the outcome of competitive fertilization, strengthening or reducing the initial differences in intrinsic sperm quality among competing males. We used a balanced split clutch design, in which an egg batch was split into two pools and fertilized by sperm from two random males in equal amount, with the only difference among the two sub-clutches being whether FRF was added or absent. In each replicate we collected the eggs and the FRF from one female and the ejaculates from two males (randomly labelled A and B), for a total of 20 replicates (unique triplets of 2 males and 1 female). After removing the FRF from the eggs, they were rinsed and split into two pools with the same number of eggs. The FRF was re-added in one of the two pools (FRF-present treatment) but not in the other (FRF-absent treatment) and then an equal number of sperm from the two males was mixed and added to the egg pools. In parallel, the sperm performance of the two males was assessed both in the presence and absence of FRF (same ejaculate and same FRF used for the IVF).

### 2.3. Gametes and FRF collection

Gametes (eggs and sperm) and the FRF were collected in the morning following an established protocol [15]. Briefly, the fish were anaesthetized in a solution of MS222 (tricaine methanesulfonate, Sigma Aldrich; 0.17 g l^−1^), rinsed with water, carefully dried in the genital area to prevent accidental activation of gametes by water, and placed under a dissecting microscope. Males were gently squeezed to release the ejaculate that was collected in a glass micro-capillary, diluted in Hank's solution (HBSS; [37]), and maintained in ice until used (within an hour from collection). The number of sperm from each ejaculate was estimated with an automated cell counter (LUNA™) and diluted accordingly for subsequent use for IVFs and CASA analysis. Females were gently squeezed in the abdominal area to release eggs (that are released along with the FRF) on a glass slide. The FRF was collected with a Drummond micropipette (see [24]) and maintained in ice until use (within an hour from collection). The eggs were rinsed with a 0.5% solution of Bovine Serum Albumin (pH 8), which allows maintaining eggs in an inactivated state without compromising egg quality [38]. Fin clips from the caudal fin of all adults were collected and preserved in absolute ethanol for subsequent paternity analyses.

### 2.4. *In vitro* fertilizations

IVFs were performed following Pinzoni *et al*. [15]. In short, the eggs obtained by each female were divided into two pools (average egg number per pool: 33.2 ± 1.3, with the same number of eggs used in the two pools obtained from the same female), and then activated with freshwater in the FRF-absent treatment or with freshwater + FRF (final concentration of FRF: 10%) in the FRF-present treatment. An equal amount of sperm from the two competing males was mixed in an Eppendorf tube, activated with freshwater (1 : 5 sperm-water proportion) and added immediately to the egg pools. After fertilization, eggs were incubated at 28°C and checked at 7 hpf (hours post fertilization) to assess fertilization success (mean egg fertilized per pool: 21.65 ± 1.2). Embryos were collected at 30 hpf and preserved in absolute ethanol for paternity analyses.

### 2.5. Assessment of sperm performance

For each assay of sperm quality, 0.5 µl of ejaculate were transferred into a chamber on a 4-chamber slide (Leja) and activated in 2.5 µl of freshwater or 2.5 µl of the freshwater + FRF solution in randomized order. Sperm quality from each male was assessed in both conditions using a CEROS Sperm Tracker (Hamilton-Thorne Research, Beverly, MA), from which we obtained the following parameters: sperm average path velocity (VAP), straight line velocity (VSL), curvilinear velocity (VCL), linearity of the sperm trajectory (LIN, measure of path curvature), and motility (proportion of motile cells). For each sample, sperm quality was assessed on an average of 299.6 ± 7.1 SE sperm cell tracks. Sperm longevity was measured as the time from activation until ≥80% of sperm in the field of view were not motile [24].

### 2.6. Paternity analysis

We obtained a total of 808 embryos from 40 fertilization trials, from 20 unique male-female combinations (40 males and 20 females). Genomic DNA was extracted following Meeker *et al*. [39]. All individuals were genotyped at five microsatellite loci (GenBank accession numbers: Z4830, Z20450, Z11496, Z9230, Z1233) in multiplex PCRs (details on the PCR protocols can be found in [15]). The amplified fragments were separated by electrophoresis on an ABI 3100 genetic analyser (ABI PRISM, Applied Biosystems, https://www.bmr-genomics.it). Microsatellites were scored using the software Geneious v. 8.1.9 (https://www.geneious.com) and paternity was assigned using the software Cervus v. 3.0 [40] with 95% strict confidence. We were able to assign paternity to 803 out of the 808 analysed (a success rate of over 99%).

### 2.7. Statistical analyses

Statistical analyses were performed using R v. 4.2.0 [41] and JMP v. 17.0. P values of the fixed effect in mixed models were calculated by Type II Wald chi-square tests using the ‘Anova’ function of the ‘car’ package [42]. Residuals of the models were visually inspected with the Q-Q plots to check model assumptions. To check whether binomial models were overdispersed we used the ‘dispersion_glmer’ function from the *blmeco* package [43]. There was no need to correct for overdispersion in our models (fertilization rate: 0.84, paternity: 1.01). Data are reported as means ± SE.

#### 2.7.1. Principal component analysis on sperm motility traits

The various components of sperm performance were combined using principal components analysis to avoid multiple testing of correlated parameters and to better represent the selection on sperm traits acting in combination (see [44]; correlation matrices for sperm performance traits are presented in electronic supplementary material, table S1 of the supplementary materials). Note that using the separated components yielded qualitatively similar results (results in the electronic supplementary material, §2).

#### 2.7.2. Effect of the presence of FRF on fertilization rate

Fertilization rate in presence or absence of FRF was tested using a generalized linear mixed effects model (‘glmer’ function of the *lme4* package; [45]) with a binomial error distribution. The proportion of fertilized eggs was fitted as the response variable using the cbind function (cbind(successes, failures)). Family ID was added as a random factor to account for the non-independence of the data (due to the split clutch design).

#### 2.7.3. Predictors of paternity share in presence and in absence of FRF

Paternity share was analysed using two generalized linear mixed effects models (‘glmer’ function of the *lme4* package; [45]) with a binomial error distribution, one for the condition ‘presence of FRF’ and one for the condition ‘absence of FRF’. The proportion of offspring sired by male B (randomly labelled) was fitted as the response variable (using cbind function: cbind(successes, failures)) and the relative differences among competing males (male B-male A) in sperm performance (PC1 and PC2) as predictors. Family ID and male ID were added as random factors to account for the non-independence of the data (due to the split clutch design).

#### 2.7.4. Linking effect of FRF on sperm traits to change in paternity share with or without FRF

To assess whether there was a direct link between the change in sperm performance caused by the FRF and the change in the resulting paternity share, we built a linear mixed model with the difference in paternity (proportion of offspring sired) in the presence of FRF minus paternity without FRF as the dependent variable, and the difference in sperm performance (PC1, PC2) measured in FRF minus that measured without FRF as a fixed factor. Both sperm performance and paternity in presence and absence of FRF were used as percent changes based on the baseline in water (no FRF) as follows: [(sperm performance in FRF—sperm performance in water)/sperm performance in water] *100. To deal with negative numbers in the PCs of sperm performance, a fixed value (+5) was added to all values. Family ID and male ID were added as random factors.

## 3. Results

### 3.1. Principal component analysis

The principal components analysis on sperm performance traits returned two PCs with eigenvalues greater than 1 that combined explained >77% of the total variance in sperm performance. Those two PCs were used for analysis. Sperm PC1 is determined mostly and positively by sperm velocity, sperm motility and longevity, while sperm PC2 is determined positively by sperm trajectory (linearity) and beat cross frequency (table 1 for details on loadings of the two PCA and percentage of variance explained, and electronic supplementary material, table S3 for the other PCAs with eigenvalues less than 1).

**Table 1.** PCA results of sperm motility parameters. Loadings, percentage of variance explained, and eigenvalues are provided for the two main PCs.

sperm traits	PC1	PC2
*VAP (average path velocity)*	0.478	0.105
*VSL (straight line velocity)*	0.460	0.280
*VCL (curvilinear velocity*	0.480	0.021
*BCF (beat-cross frequency)*	−0.162	0.585
*LIN (path Linearity)*	0.000	0.723
*motility*	0.387	−0.208
*longevity*	0.391	−0.036
*percentage of variance explained*	57.915	20.070
*eigenvalue*	4.054	1.405

### 3.2. Effect of the presence of FRF on fertilization rate

**Figure 1.**
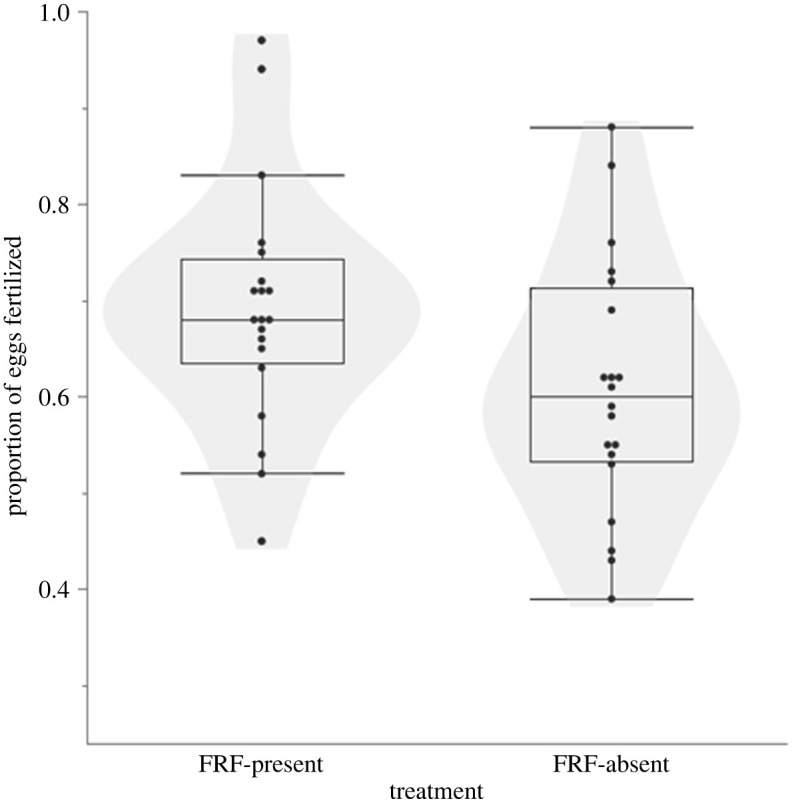
Differences in fertilization between *in vitro* fertilization in the presence (left) or absence (right) of FRF.

A higher successful fertilization rate was obtained when IVFs were performed in the presence of FRF compared to the FRF-absent treatment (*χ*^2^ = 10.736, *p* = 0.001). The average proportion of fertilized eggs was 0.69 ± 0.03 in the presence of FRF, and 0.61 ± 0.03 in the absence of FRF (figure 1).

The change in the proportion of fertilized eggs from presence to absence of FRF spanned from −0.04 to +0.24, with only two clutches showing a higher proportion of fertilized eggs when the FRF was absent (see electronic supplementary material, figure S2).

### 3.3. Predictors of paternity share in the presence and absence of FRF

**Figure 2.**
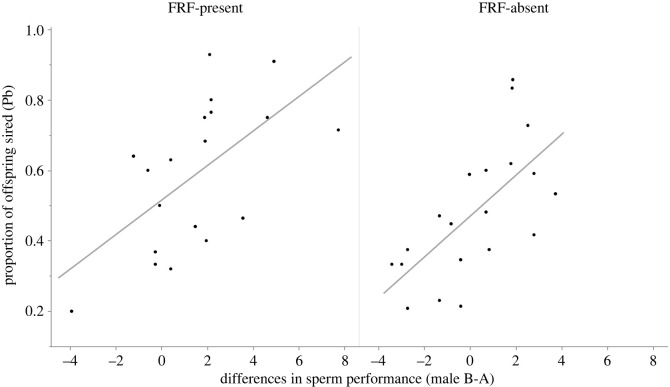
The relative differences in sperm performance (sperm PC1) between the two competing males (male B-male A) is the main determinant of paternity share both in the presence (left) and absence (right) of FRF. Each point represents a fertilization trial.

Both in the presence and absence of FRF the main predictor of paternity share was the difference in sperm velocity, motility and longevity (sperm PC1) among competing males, with males with faster and longer living sperm compared to their competitor gaining relatively more paternity (without FRF: *χ*^2^ = 15.331, *p* < 0.001, with FRF: *χ*^2^ = 8.250, *p* = 0.004, figure 2). There was no effect on paternity of the relative difference in sperm PC2 (without FRF: *χ*^2^ = 0.686, *p* = 0.408, with FRF: *χ*^2^ = 0.411, *p* = 0.521, electronic supplementary material, figure S3).

However, these relative differences in sperm PC1 among competing males, predicting paternity share in both FRF absence and presence, were often different (in direction and magnitude) across these two conditions, due to the differential effect of the FRF on sperm performance (see electronic supplementary material, figure S4 and next section).

### 3.4. Linking effect of FRF on sperm traits to change in paternity share with or without FRF

On average, the difference in sperm performance (sperm PC1) from the FRF-absent to the FRF-present treatment was 28.03%, indicating that FRF on average increased sperm performance compared to when sperm performance was measured in the absence of FRF. However, this was not always a positive effect, as in some cases the effect of FRF on sperm performance was negative (range of FRF effect, min: −49.5%, max: +148.4%). The difference between sperm performance with or without FRF was significantly different from zero (one sample *t*-test: *t* = 3.783, *p* < 0.001, 95% CI: 13.04–43.03).

**Figure 3.**
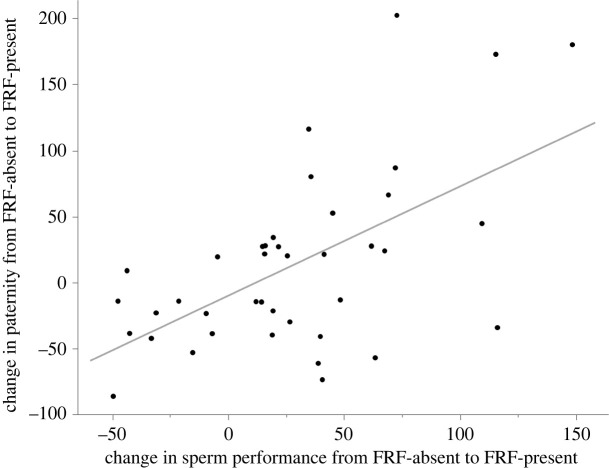
Change in the paternity success in relation to the change in sperm performance (PC1) caused by FRF. Changes are expressed in % from the value obtained in the presence of FRF minus the value obtained in the absence of FRF. As an example, a change of +69% in sperm performance from FRF-absent to FRF-present resulted in a change in the paternity success of +66%. Each data point represents a male (*N* = 40).

The model investigating whether the change in paternity share was associated with the change in sperm performance revealed a significant positive association between paternity change (in % from FRF-absent to FRF-present treatment) and sperm PC1 but not sperm PC2 (sperm PC1: *χ*^2^ = 19.056, *p* < 0.001, sperm PC2: *χ*^2^ = 1.869, *p* = 0.172). Analysing it as a simple correlation among the change in sperm PC1 and change in paternity also resulted in a significant positive correlation (*r* = 0.58, *p* < 0.001, figure 3), while no significant correlation emerged among the change in sperm PC2 and the change in paternity (*r* = −0.21, *p* = 0.196).

## 4. Discussion

With this study, we provide novel and critical insights into mechanisms and consequences of the role of female reproductive fluid in post-mating fertilization dynamics. Our results indicate that the effect of FRF on sperm performance alters sperm competition dynamics and the ensuing paternity share, affecting the fitness landscape of competing males. Moreover, we reveal that the FRF effect is directly related in magnitude and direction to the shift in paternity caused by the presence of FRF at fertilization. Our study therefore provides further evidence of the crucial role of FRF in fertilization and post-mating sexual selection processes but extends previous work by directly linking the change in paternity caused by the FRF with its phenotypic effect on sperm traits. Our main results are that (i) overall fertilization rate is higher in the presence of FRF, (ii) relative differences in sperm velocity among competing ejaculates constitute a reliable predictor of paternity share in both FRF-absent and FRF-present treatments but (iii) such relative differences change from absence to presence of FRF due to the impact of FRF on sperm performance (current study; [24]), and (iv) the change in paternity share is predicted in magnitude and direction by the effect of FRF on sperm performance.

### 4.1. Effect of the presence of FRF on fertilization rate

Fertilization rate was higher in the presence of FRF, confirming that the presence of FRF contributes to a successful fertilization, mimicking the natural spawning environment of the species. This result is likely attributable to multiple interlaced effects, including the generalized positive effect of FRF on sperm traits (this study, and [24]), egg viability, and fertilization window [15].

### 4.2. Predictors of paternity share in the presence and absence of FRF

We showed that in both the absence and presence of FRF, the relative difference in sperm performance among competing males was the main predictor of paternity share, with males producing faster and longer-living sperm siring relatively more offspring than their competitor. This is an important step forward in the knowledge of fertilization dynamics in this species, as it is the first evidence that sperm performance is a proxy for male competitive fertilization ability in the zebrafish. Despite being a very well-known model organism, zebrafish studies on mating systems and sexual selection have been lagging behind, and little is currently known about both pre- and post-mating selective dynamics in this species. Here, we showed that the relative differences in sperm swimming velocity, longevity and motility among rival males are the best predictors of paternity success, corroborating the findings of previous studies demonstrating that sperm velocity is the main determinant of male competitive fertilization success in fishes (e.g. [46–50]).

Clearly, differences in sperm number between competing males are also likely to contribute to the variance in paternity success in a natural situation (as in our study sperm number was standardized). Further studies are therefore needed to elucidate the relative importance of sperm quantity and quality in this species.

### 4.3. Linking effect of FRF on sperm traits to change in paternity share with or without FRF

A recent study by Alonzo *et al.* [29] has investigated the role of FRF in the outcome of competitive fertilization by comparing paternity share in the presence or absence of FRF. That study showed how the presence of FRF can change the paternity share among competing males by affecting the relative importance of sperm number and quality. Our study, similarly, confirms that FRF during fertilization contributes significantly to the variance in male competitive reproductive fitness while also expanding our understanding of FRF's role in post-mating dynamics. In a unique approach, we standardized sperm number among rival males, thus focusing on sperm quality over quantity, and combined in the same experiment, within female and within ejaculate, sperm performance measurements and paternity distribution in the presence and absence of FRF. As such, we have, for the first time, unequivocally demonstrated the direct association between the change in sperm performance due to the FRF and the change in paternity outcome. We were therefore able to simulate a situation without FRF (possibly unrealistic as FRF is always present in natural fertilization) where only sperm competition operates, i.e. only differences in the intrinsic quality of sperm between males matter, and a situation in the presence of FRF, in which sperm competition and cryptic female choice operate simultaneously. Thereby, we were able to disentangle the male from the female-driven (FRF-mediated) effects. FRF can dramatically affect the performance of sperm compared to their intrinsic baseline (measured in absence of FRF), in some cases even making sperm swim twice as fast. Our key result is that the direction and magnitude of the effect of FRF on sperm performance predicts the ensuing change in paternity share between the two males from FRF-absent to FRF-present fertilizations. The influence of FRF on sperm performance was mainly positive, but its magnitude showed large variation depending on the specific male-female combination, as previously found in [24]. Interestingly, there were some cases (11 out of 40, 27%) in which the effect of FRF on sperm performance was even negative (meaning that sperm swam slowly in the FRF-present compared to FRF-absent), and in almost all those cases (9 out of 11) the change in paternity from FRF-present to FRF-absent was also negative (meaning that the male fertilization success dropped from FRF-absent to FRF-present IVFs). Our results indicate that the change in sperm performance from FRF-absent to FRF-present serves as an indicator of female post-mating preference for specific males, resulting in a fertilization advantage towards the preferred male. It remains to be determined what makes one male preferred over another. In other species, FRF bias fertilization towards unrelated, or genetically compatible, partners [27,28,30]. Based on the results of our current study and considering that the pre-mating female choice of this species seems to be based on genetic compatibility at the MHC loci (Santacà, Grapputo, Devigili & Gasparini in prep [51]), it is likely that the cryptic female choice mechanism we report here is also linked to genetic compatibility. The change in paternity share detected across treatments (absence/presence of FRF) could therefore indicate a cryptic female choice mechanism mediated by the FRF to favour the most genetically compatible partner, but this aspect remains to be tested directly. Our combined results constitute the first evidence of a direct link between the effects of FRF on sperm performance and its influence on the paternity share of competing males, effectively showing how FRF influences fertilization dynamics by acting on the main predictors of male post-mating success, sperm swimming velocity, motility and longevity.

In a recent study, we showed that FRF increases multiple paternity and the opportunities for post-mating sexual selection, by prolonging the egg fertilization window [15]. Here, we demonstrated that, by affecting male competitors' sperm performance, FRF can effectively bias competitive fertilization outcomes, providing a mechanism by which females can exert cryptic female choice during the time (albeit brief) between gamete release and fertilization. Somewhat surprisingly, direct evidence of cryptic female choice remains limited, especially for external fertilizers. As such, our results constitute an important step forward in our understanding of the widespread occurrence of cryptic female choice and the mechanisms underlying this process [10]. This is especially true in external fertilizers where pre-mating sexual selection may be limited and where sexual selection at post-mating stage is expected to be stronger than in internal fertilizers, due to the very long evolutionary history shaping sperm competition and cryptic female choice in these species [52]. For this reason, caution is necessary when generalizing these mechanisms across organisms with internal and external fertilization. In the context of internal fertilization, females possess diverse mechanisms to regulate the fertilization process (as reviewed by [10] and [7]), alongside the influence of female reproductive fluid (FRF). Conversely, among external fertilizers, cryptic female choice mechanisms are more limited and therefore FRF may represent the primary mechanism at play. In conclusion, our analysis reveals a direct link and concordant pattern of FRF effects on sperm quality and competitive fertilization outcome. This study therefore unambiguously demonstrates that the FRF directly mediate post-mating female control through its differential effect on sperm performance and that the sole effect on sperm quality is sufficient to predict the magnitude of the fitness (paternity share) effects. FRF mediating cryptic female choice by manipulating sperm performance is likely to be taxonomically widespread, and occurring across a diversity of mating systems, and our work emphasizes how important it is to consider the female contribution to the fertilization environment when investigating post-mating processes [11].

## Data Availability

All data supporting this study have been deposited in figshare: https://doi.org/10.6084/m9.figshare.24412519.v1 [53]. The data are provided in electronic supplementary material [54].
